# Advances in Traditional Chinese Medicine for Modulating DNA Methylation in the Treatment of Inflammatory Diseases

**DOI:** 10.3390/ijms26136331

**Published:** 2025-06-30

**Authors:** Cui Zhang, Chonkit Lio, Nana Li, Cong Huang, Xueming Yao, Jinfang Luo

**Affiliations:** 1 Department of Basic Medicine, Department of Pharmacy, Key Laboratory on the Property & Effect of Chinese Medicine (Ethnic Medicine), Guizhou Genuine Herbs Center of Consistency of Utility, The Provincial Key Miao Medicine Laboratory of Guizhou, Guizhou University of Traditional Chinese Medicine, Guiyang 550025, China; 18419261387@163.com (C.Z.); 18085483692@163.com (N.L.); huangcong236@163.com (C.H.); 2 Faculty of Chinese Medicine, Macau University of Science and Technology, Avenida Wailong, Taipa, Macau, China; 3 State Key Laboratory of Quality Research in Chinese Medicine, Macau University of Science and Technology, Avenida Wailong, Taipa, Macao, China

**Keywords:** traditional Chinese medicine, DNA methylation, inflammatory diseases, epigenetic mechanism

## Abstract

DNA methylation is a crucial epigenetic mechanism that modifies the epigenome without altering the DNA sequence, leading to heritable changes in gene expression and playing a vital role in biological processes. The regulation of DNA methylation has gained significant attention in recent years for its role in inflammatory diseases, with numerous studies indicating a correlation between abnormal gene methylation and disease development. However, current research on mature methylation-regulation drugs remains in its infancy. Traditional Chinese medicine (TCM) has been demonstrated to have a potential therapeutic effect in treating inflammatory diseases by modulating DNA methylation. In this review, we provide an overview of how DNA methylation participates in inflammatory diseases and how TCM assesses its function in regulating DNA methylation modifications. We aim to demonstrate a theoretical foundation for further research on the therapeutic targets and mechanisms of TCM in inflammatory disease treatment.

## 1. Introduction

Inflammatory diseases encompass a range of conditions, including rheumatoid arthritis, inflammatory bowel disease, and chronic atrophic gastritis (CAG). The pathogenesis of inflammatory disease is complex, involving multiple processes during inflammation, such as immune cell activation, cytokine imbalance, and oxidative stress [1]. In recent years, increasing evidence has highlighted the importance of epigenetic regulation in mediating these inflammatory responses. DNA methylation is one of the earliest discovered and most extensively studied epigenetic modifications. The process of DNA methylation is catalyzed by DNA methyltransferases (DNMTs); a methyl group is added to the 5th carbon of cytosine in Cytosine-phosphate-Guanine (CpG) dinucleotides within the genome, resulting in the formation of 5-methylcytosine (5-mC) [2].

While modern biomedical research is steadily revealing the molecular basis of inflammatory diseases, traditional medical systems like TCM contribute a holistic approach rooted in centuries of clinical experience and observation. TCM, Japanese–Chinese medicine, and Korean Sasang constitutional medicine have common origins; all are valuable components of conventional medicine and contain a diverse array of active compounds that can modulate inflammatory responses through multiple pathways [3]. In recent years, an increasing number of studies have demonstrated that TCM has the potential to regulate DNA methylation, providing new therapeutic strategies for inflammatory diseases. To date, there has been a lack of comprehensive review articles on the regulation of DNA methylation by TCM in the treatment of inflammatory diseases. This review aims to address this gap by synthesizing and analyzing recent studies on the topic. We conducted an extensive review of many domestic and international research related to the interplay between TCM, DNA methylation, and inflammatory diseases. Given our focus on TCM—a traditional Chinese therapeutic modality—and its regulatory effects on DNA methylation in inflammatory diseases treatment—we found that a substantial portion of relevant literature is published in Chinese. Accordingly, we have cited a significant number of Chinese references. This review provides readers with an in-depth understanding of the mechanisms through which TCM regulates DNA methylation and offers a comprehensive analysis of the TCM–DNA methylation–inflammatory disease triad.

## 2. Role of DNA Methylation in Inflammatory Diseases

### 2.1. Role of DNA Methylation in Joint and Skeletal Inflammatory Diseases

#### 2.1.1. Rheumatoid Arthritis

Rheumatoid arthritis (RA) is a chronic autoimmune disorder characterized by persistent synovial inflammation, which may ultimately result in joint deformities and functional impairment [4]. Accumulating evidence suggests that DNA methylation plays a crucial role in the pathogenesis of RA. Altered methylation patterns have been observed in peripheral blood mononuclear cells (PBMCs), T cells, B cells, and other immune cells from patients with RA [5]. Fibroblast-like synoviocytes (FLS) play a key role in the development of synovial lesions and joint destruction in RA. Notably, the DNA of RA-FLS exhibits widespread hypomethylation [6]. In addition, the promoter region of the chemokine ligand gene C-X-C motif chemokine ligand 12 *(CXCL12)* is also hypomethylated in RA-FLS [7]. CD4^+^ T cells, another key cell type implicated in RA pathogenesis, show similar epigenetic changes. In early RA patients, the promoter region of the *TNF-α* gene in naïve CD4^+^ T cells is hypomethylated [8], and the promoter of the *IL-8* gene is also hypomethylated in CD4^+^ T cells from RA patients [9]. Pharmacological studies have highlighted the importance of DNA methylation in RA; treatment with 5-azacytidine (5-Aza), a DNMTs inhibitor, remarkably reduces the expression of multiple inflammatory cytokines in RA mouse FLS, thereby inhibiting disease progression [10]. Moreover, Svendsen et al. suggested that conventional disease-modifying antirheumatic drugs (DMARDs), such as methotrexate, can reverse the hypomethylation of the promoter region of the inflammation-associated gene AGPAT1, restoring its hypermethylated state [11]. Collectively, these findings suggest that abnormal DNA methylation is closely linked to the pathogenesis of RA.

#### 2.1.2. Osteoarthritis

Osteoarthritis (OA) is a prevalent orthopedic disorder driven by synovial inflammation, chondrocyte apoptosis, and extracellular matrix degradation, ultimately leading to osteophyte formation and cartilage destruction [12]. DNA methylation is closely associated with the onset and progression of OA. Studies have shown that catabolic genes in OA chondrocytes are often hypomethylated, whereas anabolic genes tend to be hypermethylated [13]. Methylation at specific promoter regions or within genes can regulate gene transcription and expression in OA chondrocytes [14]. DNA methyltransferase 3B (DNMT3B) plays a key role in maintaining the function and stability of articular chondrocytes. It is highly expressed in healthy articular cartilage. Still, its expression declines with age, leading to reduced epigenomic stability, impaired metabolic function of chondrocytes, and the promotion of inflammatory pathological features [15]. Zhu et al. suggested that elevated levels of DNA methyltransferase 1 (DNMT1) and DNA methyltransferase 3A (DNMT3A) in both mouse and human OA cartilage lead to hypermethylation of the peroxisome proliferator-activated receptor gamma (*PPARγ*) promoter, resulting in suppressed *PPARγ* expression [16]. Inhibition of DNMT1 and DNMT3A using 5-Aza in destabilization of the medial meniscus (DMM) mouse models reversed *PPARγ* promoter methylation, restored PPARγ expression, and alleviated cartilage destruction.

#### 2.1.3. Gouty Arthritis

Gouty arthritis (GA) is an autoinflammatory disease characterized by the deposition of monosodium urate (MSU) crystals in joints and surrounding tissues, often associated with disorders of purine metabolism and impaired uric acid excretion [17]. Recent studies have found that the onset of gout is closely related to abnormal DNA methylation. Elevated methylation levels of the *UMOD* gene have been observed in patients with gout compared to healthy controls [18]. In contrast, the promoter regions of *NRBPI* and *CCL2* exhibit reduced methylation [19]. Peng et al. detected the mRNA expression levels of *DNMT1*, *DNMT3A*, and *DNMT3B* in PBMCs from patients with primary gout and healthy individuals [20]. Their results showed that *DNMT1* and *DNMT3A* expression were significantly lower in both the acute gout (AG) group and the intermittent gout (IG) group compared to the healthy control group, with *DNMT3A* expression being further reduced in the AG group relative to the IG group. No significant difference was found in *DNMT3B* expression among the three groups. These findings suggest that *DNMTs* play an important role in the pathogenesis of primary gout.

### 2.2. Inflammatory Disorders of the Digestive System

#### 2.2.1. Chronic Atrophic Gastritis

Chronic atrophic gastritis (CAG) is a persistent digestive system disorder characterized by atrophy and loss of intrinsic glands in the gastric mucosa, thinning of the mucosa layer, and thickening of the muscularis mucosal [21]. Abnormal DNA methylation plays a crucial role in the development of CAG and its associated carcinogenesis. In patients with chronic gastritis, the inflammatory response in the gastric mucosa leads to widespread hypermethylation and hypomethylation of numerous CpG sites [22]. Accumulating evidence has shown that DNA methylation levels in patients with gastritis correlate with the degree of gastric mucosal inflammatory activity. In gastric precancerous lesions, such as CAG with intestinal metaplasia, high levels of promoter methylation have been found in tumor-related genes. During carcinogenesis, the DNA methylation level in the CpG island region increases gradually, leading to the loss or inactivation of tumor suppressor genes in the specific areas, which promotes the occurrence and development of gastric cancer [23]. Moreover, Guo et al. found that the DNA methylation status affects the development of gastric mucosal atrophy in patients with chronic gastritis, with those exhibiting atrophy showing elevated DNA methylation levels [24].

#### 2.2.2. Ulcerative Colitis

Ulcerative colitis (UC) is a chronic inflammatory bowel disease characterized by persistent damage to the colonic epithelial mucosa, which represents a key pathological feature of the condition [25]. Accumulating evidence suggests that abnormal DNA methylation is strongly associated with the pathogenesis of UC [26]. DNA hydroxy methylase 10–11 translocation protein 2 (TET2), a key dioxygenase responsible for DNA hydroxy methylation, has been demonstrated to regulate interleukin-6 (IL-6) levels and attenuate dextran sulfate sodium (DSS) - induced colonic inflammation in UC colon [27]. Feng et al. further found that the loss of the *TET2* gene leads to abnormal DNA methylation in the colon and exacerbated inflammation in the colon. Moreover, they found that acupuncture alleviated colonic inflammation in UC by regulating *TET2* expression. Additionally, both mild moxibustion and electroacupuncture reduced the pathological overexpression of 5-mC in UC, with mild moxibustion resulting in a more pronounced reduction compared to electroacupuncture [28].

### 2.3. Inflammatory Disorders of the Respiratory System

#### 2.3.1. Allergic Rhinitis

Allergic rhinitis (AR) is a common respiratory disease and a non-infectious chronic inflammatory disease of the nasal mucosa, primarily mediated by immunoglobulin E (IgE) [29]. DNA methylation plays a crucial role in the pathogenesis of AR [30], as it regulates gene transcription levels, increases the risk of allergic airway diseases, regulates AR symptoms, and contributes to immune homeostasis. Studies have found that regulating DNA methylation can improve airway hyperresponsiveness and eosinophil (EOS) infiltration, reduce IgE levels, and alleviate allergic symptoms in AR rat models [31]. Jia et al. established an SD rat model of AR to study the regulatory effect of demethylating agent 5-Aza, a DNA methyltransferase inhibitor, on the expression of *DNMT1*. Their results indicated that 5-Aza influences the onset and development of AR by regulating *DNMT1* expression [32]. Morin et al. reported that the composition of upper respiratory tract microbes in infancy is involved in the development of childhood AR, partially mediated through altered DNA methylation in upper respiratory mucosal epithelial cells [33]. Zhou et al. used a high-throughput methylation microarray to identify differentially methylated genes in inferior turbinate tissue samples from AR patients, suggesting that gene methylation may be a key contributor to AR pathogenesis [34].

#### 2.3.2. Pneumonia

Pneumonia is a prevalent lower respiratory tract infection and a significant contributor to global health issues [35]. Abnormal DNA methylation has been implicated in the immune dysregulation associated with pneumonia, influencing disease outcomes by regulating the functions of various immune cells. Macrophages, as key innate immune cells in pulmonary infections, have their differentiation, development, and functional activities closely regulated by DNA methylation processes [36]. Ampomah et al. indicated that methionine inhibits lipopolysaccharide-induced lung inflammation by increasing DNA methylation levels in macrophages [37]. Singer et al. found that the increased number of Tregs in the lungs of mice that treated with DNA methyltransferase inhibitors and lung inflammation was effectively alleviated in an influenza virus-induced pneumonia mice model [38]. Furthermore, Cole et al. reported that prenatal exposure to tobacco smoke in mice may elevate the risk of lung inflammation in offspring by altering the DNA methylation pattern in pulmonary tissue cells [39].

### 2.4. Inflammatory Disorders of the Cardiovascular System

#### Atherosclerosis

Atherosclerosis (AS) is a chronic inflammatory condition of the vascular wall, characterized by lipid metabolism disorders such as cholesterol accumulation and macrophage-mediated lipid uptake [40]. DNA methylation plays an important regulatory role in the initiation and progression of AS [41]. Altered DNA methylation patterns—either at specific CpG sites or across the genome—have been detected in atherosclerotic plaques and peripheral blood cells of patients [42]. Dysregulated DNA methylation contributes to endothelial dysfunction, macrophage-driven inflammation, abnormal proliferation of vascular smooth muscle cells (VSMCs), plaque rupture, and thrombosis, thereby exacerbating AS pathology [43]. Krüppel - like factor 4 (*KLF4*) plays a crucial role in AS development. Jiang et al. reported that disturbed blood flow induces the enrichment of DNMT3A on the *KLF4* promoter, leading to DNA methylation of the CpG island in the *KLF4* promoter, thereby inhibiting *KLF4* transcription and weakening its inhibitory effect on AS inflammation. Blocking upstream *KLF4* methylation was shown to ameliorate the pro-inflammatory and pro-thrombotic effects caused by disturbed blood flow [44,45]. Similarly, Tang et al. reported that DNMT1 expression was increased in macrophages within AS plaque and that DNMT1 promotes AS-associated inflammation by mediating DNA methylation of the *KLF4* promoter region and suppressing its expression [46].

### 2.5. Inflammatory Diseases of the Skin

#### 2.5.1. Psoriasis

Psoriasis (Ps) is an immune-related, chronic inflammatory skin disease [47]. Growing evidence supports that DNA methylation is closely related to the onset of Ps [48]. For instance, Zhang et al. indicated that DNA methylation level was increased in the skin lesions and PBMCs in Ps patients. Notably, the Ps Area and Severity Index (PASI) score was positively correlated with the degree of DNA methylation but not with the methylation level of PBMCs [49]. In 2013, Zhang et al. reported that the number of high-methylation differentially methylated regions was significantly higher than that of low-methylation differentially methylated regions in Ps patients’ skin samples [50]. Similarly, Zhang et al. found that TET3 and KLF4 levels were significantly upregulated in the Ps patients’ skin lesions, and the DNA methylation level of the *KLF4* promoter was significantly downregulated [51]. In another study, Gu et al. analyzed the genomic DNA methylation profiles of epidermal cells in lesion skin samples from 12 Ps patients before and after ultraviolet B treatment and found that a total of 3665 methylation variable positions were overall hypomethylated in Ps patient samples. Notably, the DNA methylation pattern was reversed after 2 to 3 months of treatment, demethylation occurred in the Ps patients’ skin samples and the patients’ condition improved, indicating that DNA methylation is a dynamic and is reversible process in Ps patients [52]. Taken together, these findings illustrate that DNA methylation is not only a key epigenetic factor in the pathogenesis of Ps but also a dynamic and reversible process.

#### 2.5.2. Acne Vulgaris

Acne, more common on the face, is a chronic inflammatory disease of the pilosebaceous gland unit, characterized by comedones, papules, pustules, nodules, cysts, and scar formation [53]. DNA methylation and epigenetics play an important role in acne inflammation and immunity. For example, Liu et al. found that acne in the early stage was closely associated with 31,134 differentially methylated sites and 770 differentially methylated genes [54]. Similarly, Xiao et al. indicated 67,984 differentially methylated sites on different chromosomes, involving 4635 differentially methylated genes, by using Illumina Infinium Methylation EPIC Bead Chip technology and screened for abnormal methylation of the whole genome DNA of acne patients [55]. These findings suggest abnormal DNA methylation may be one of the causes of acne and play a crucial role in which epigenetics regulate the pathogenesis of acne. Likewise, Wang et al. also found 275 differentially methylated sites and 194 differentially methylated genes via detecting blood DNA samples from patients with severe acne and healthy controls by using Illumina Infinium Methylation EPIC Bead Chip [56]. They found that the difference in whole-genome DNA methylation between the two was statistically significant, suggesting that abnormal DNA methylation may be involved in the onset and development of severe acne. Together, these studies suggest that abnormal DNA methylation is a critical epigenetic factor in the pathogenesis of acne, influencing inflammation and immune responses in affected individuals.

### 2.6. Systemic Inflammatory Disorders

#### Systemic Lupus Erythematosus

**Figure 1:**
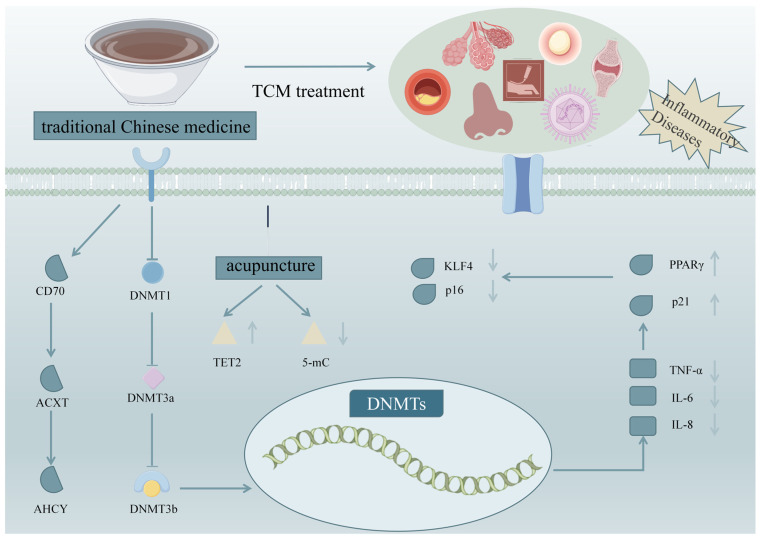
The mechanism of TCM regulating DNA methylation in the treatment of inflammatory diseases (Note: ↑ denotes up-regulated genes, ↓ denotes down-regulated genes).

Systemic lupus erythematosus (SLE) is an autoimmune-mediated diffuse connective tissue disease characterized by immune inflammation [57]. Recently, epigenetics research on SLE has gained significant attention, with accumulating evidence suggesting the role of DNA methylation in its pathogenesis [58]. For example, Zhao et al. indicated that DNA hypomethylation in SLE patients leads to the abnormal activation of CD4^+^ T cells, which in turn stimulates B cells to secrete excessive immunoglobulins, finally resulting in autoimmune disorders [59]. Similarly, Zhu et al. reported that SLE patients exhibited significantly reduced DNA methylation levels, which were positively correlated with the SLE-Disease Activity Index (SLE-DAI), indicating the important role of epigenetic changes in disease progression [60]. Expanding on these findings, Zhao et al. observed that the methylation level of two CG base pairs in the *IFI44L* promoter was significantly decreased in SLE patients, suggesting a gene-specific methylation pattern associated with SLE [61]. Similarly, Nawrocki et al. revealed that the transcription levels of *DNMT1* and *DNMT3A* were lower in SLE patients compared to healthy controls. Notably, *DNMT1* transcription levels were positively correlated with SLE-DAI, while *DNMT3A* transcription was negatively correlated with patient age [62]. Furthermore, Wang et al. suggested that reduced DNA methylation in SLE patients was associated with enhanced activity of the catalytic subunit of protein phosphatase 2A (PP2Ac) and that this epigenetic regulation was dynamically influenced by ERK pathway phosphorylation [63]. Taken together, these studies provide compelling evidence that DNA methylation is a key epigenetic mechanism underlying the onset and progression of SLE. This epigenetic regulation not only influences immune cell activation but also modulates gene expression, highlighting its potential as a therapeutic target for SLE management. The mechanism of TCM in the treatment of inflammatory diseases by regulating DNA methylation is shown in Figure 1. As shown in Figure 1, TCM can treat RA, CAG, UC, AR, AS, acne, SLE, and other inflammatory diseases by regulating DNA methylation-related proteins. However, the existing research is not sufficiently detailed. From the perspective of methylation-related proteins, there is a lack of research at a deeper level, specifically regarding which pathway regulates and how it controls the specific mechanism of action. Exploring various pathways to gain a deeper understanding of how TCM regulates DNA methylation in the treatment of inflammatory diseases to provide a more scientific foundation for clinical therapies used is necessary in the future.

## 3. Examples of Studies on Different Classes of Traditional Chinese Medicines Modulating DNA Methylation to Treat Inflammatory Diseases

### 3.1. Compound Traditional Chinese Medicine Formula

Numerous studies have demonstrated that TCM formulations modulate DNA methylation and have shown that DNA methylation exerts therapeutic effects in various disease models. For instance, He et al. found that Xiaopi granules can treat gastric mucosal dysplasia in rats with CAG by downregulating the expression of the *DNMT3B* gene [64]. In another study, Chen et al. found that Liuwei Dihuang pills may prevent plaque formation and endothelial cell apoptosis in postmenopausal atherosclerotic (AS) mice by inhibiting DNMT1 expression and *ER-α* gene methylation [65]. Further exploring the regulatory impact of TCM on DNA methylation, Zhao et al. found that the combined use of Xuefu Zhuyu capsule and Siji Sanhuang capsule may stabilize AS plaques by increasing the overall DNA methylation and DNMT levels in the serum of AS mice [66]. In line with this, Zhang et al. indicated that the optimized Shenyuandan formula may improve insulin resistance and resist AS by reducing the gene methylation and DNMT1 levels in the serum of AS mice [67]. Moreover, Ren et al. showed that Danggui Shaoyao San improved the blood lipid level and plaque area in AS mice by inhibiting the serum methylation level and the expression of DNMT1 in AS plaques [68]. Zhou Xuelei found that the ginseng and astragalus compound may regulate the DNA methylation modification of certain specific genes to reduce the inflammatory damage of vascular endothelial cells and play a role in protecting vascular endothelial cells and play a role in reducing the plaque area in AS mice [69]. Liu et al. investigated the effect of Wutou decoction on DNA methylation in CIA rats. The results showed that compared with the normal group, the expression of DNMT1 mRNA in the model group rats was high; compared with the model group, the expression of *DNMT1* mRNA in the drug group rats was significantly reduced, suggesting that Wutou decoction may inhibit the expression of *DNMT1* mRNA, inhibit rat synovial hyperplasia, and alleviate arthritis symptoms [70]. Chen et al. found in an experimental study that Baihu Jia Guizhi decoction mainly regulates the expression levels of methylation up-regulated gene *ACXT* and methylation down-regulated genes *AHCY* and *RPL3* to control the generation and release of inflammatory factors, thereby significantly improving the swelling degree and pathological damage of the feet of rats with heat arthritis model [71]. Sun et al. found that the serum containing the Lang-Chuang-Ding decoction could upregulate the methylation level of the *CD70* gene promoter in peripheral blood monocytes of female patients with SLE and inhibit the expression of the *CD70* gene in patients with SLE, thereby exerting a therapeutic effect [72]. Together, these studies demonstrate that TCM formulations can effectively modulate DNA methylation, providing a potential epigenetic mechanism for their therapeutic effects across various disease models. The treatment of inflammatory diseases by the Chinese herbal compound mentioned above is shown in tabular form in Table 1.

Table 1 briefly summarizes the treatment examples of different TCM compounds on inflammatory diseases by regulating DNA methylation, including Xiaopi granules, through the rat model of CAG, based on the target of regulating DNA methyltransferase DNMT3B, to treat chronic atrophic gastritis. The Liuwei Dihuang pill plays a role in the treatment of AS based on the regulation of *DNMT1* and *ER-α* gene methylation levels through an AS mouse model. The combined application of Xuefu Zhuyu capsule and Siji Sanhuang capsule, as demonstrated in the AS mouse model, regulates DNMT level and plays a role in treating AS. Similarly, Wutou decoction plays a role in the treatment of arthritis by targeting DNMT1 in the CIA rat model. On the other hand, Baihu Jiaguizhi decoction has been shown to inhibit inflammation in heat arthralgia rat model by modulating the expression of methylation-up-regulated gene *ACXT*, as well as methylation-down-regulated gene *AHCY* and *RPL3*. Building on this, we summarize the broader role of TCM in treating inflammatory diseases through the regulation of methylation-related genes such as *DNMT3B*, *DNMT1*, *ACXT*, *AHCY*, and *RPL3*. However, current research on these mechanisms remains limited and lacks in-depth exploration. Future studies are necessary to investigate the underlying pathways to elucidate how TCM regulates DNA methylation and thereby exerts its therapeutic effect in inflammatory diseases. Such work will provide a stronger scientific foundation for its clinical application.

### 3.2. Active Ingredients of Traditional Chinese Medicine

Shu et al. explored the mechanism by which daphnetin acts against the effects of RA. Their results demonstrated that daphnetin reduced the expression of DNMT1, DNMT3A, and DNMT3B in the synovium of rats with collagen-induced arthritis (CIA), leading to the demethylation of pro-apoptotic genes, including *DR3*, programmed death gene 5, *FasL*, and *p53*. This, in turn, upregulated the expression of pro-apoptotic genes and proteins, thereby promoting the apoptosis of synovial cell [73]. Zhou et al. showed that gardenia jasminoides reduce the total cholesterol level of cells. Both low and high doses of gardenia jasminoides modulated abnormal DNA methylation in macrophage-derived foam cells, exhibiting a bidirectional regulatory effect of demethylation and methylation, thus exerting an anti-AS effect [74]. Jiang et al. demonstrated that periplogenin can inhibit DNA synthesis, downregulate proliferation-related proteins, upregulate p21 expression, and induce cell cycle arrest or programmed necrosis in HaCaT cells, thereby inhibiting cell viability and exerting a therapeutic effect on Ps [75]. Similarly, Zhang et al. found that hydroxycamptothecin reduces DNMT1 expression and downregulates the promoter methylation level of the *p16* gene promoter, thereby increasing p16 expression in PBMCs from patients with SLE [76]. In another study, Luo et al. found that Sinomenine (SIN) could upregulate the DNA methylation level of the *mPGES-1* promoter in the A549 cell line, and its DNA methylation level was negatively correlated with the expression of mPGES-1. Studies have confirmed that SIN can selectively inhibit the expression level of the *mPGES-1* gene by affecting the methylation level of the *mPGES-1* gene promoter region, thus playing a role in the treatment of inflammation [77]. The treatment of inflammatory diseases using the active ingredients of TCM mentioned above is presented in tabular form as shown in Table 2.

Table 2 briefly summarizes the treatment examples of different TCM monomer components on inflammatory diseases by regulating DNA methylation, including daphnetin through CIA rat model, based on the regulation of DNA methyltransferase DNMT1, DNMT3A and DNMT3B expression, play a role in the treatment of inflammation; geniposide inhibits inflammation by regulating abnormal DNA methylation genes through a macrophage-derived foam cell model; periplogenin plays a role in the treatment of Ps through HaCaT cells, based on the control of DNA synthesis-related enzymes; hydroxycamptothecin plays a role in the treatment of SLE by regulating the methylation of *DNMT1* and *p16* gene promoter region through peripheral blood mononuclear cells. Sinomenine inhibits inflammation by regulating the expression of DNA methylation level of *mPGES-1* gene promoter in A549 cells, and so on. We summarized the mechanism of different TCM monomer components in the treatment of inflammatory diseases by regulating the expression of methylation-related genes, such as *DNMT3A*, *DNMT3B*, *DNMT1*, *p16*, and *mPGES-1*. However, research on these mechanisms is not yet in depth. In the future, more in-depth studies of related mechanisms are expected to clarify how TCM monomer components specifically regulate DNA methylation, thereby playing their therapeutic role in inflammatory diseases and providing a more scientific basis for clinical treatment of these conditions.

## 4. Conclusions and Perspectives

In this review, we summarized recent progress in understanding the role of DNA methylation in inflammatory diseases and emphasized the regulatory potential of TCM. While notable strides have been made, continued research is crucial to unravel the underlying mechanisms and facilitate the translation of these insights into clinical applications. While progress has certainly been made, much about the underlying mechanisms remains unclear. To move from bench to bedside, more focused research is needed to connect experimental insights with real-world clinical practice. Inflammatory diseases are common and involve a range of complex factors, often resulting in a significant reduction in quality of life for affected individuals. Among the many regulatory processes involved, DNA methylation is generally linked to gene silencing, while regions lacking methylation tend to be associated with active gene transcription.

Building on this context, epigenetic regulation—particularly thorough DNA methylation—has emerged as a key mechanism in the pathogenesis of inflammatory diseases. These conditions are both complex and prevalent, often compromising a patient’s quality of life. DNA methylation is related to gene silencing, whereas the absence of methylation is generally linked to gene activation. Disruptions in normal DNA methylation patterns—whether due to environmental factors, genetic predispositions, or pathological conditions—can alter gene expression and trigger disease development through multiple interconnected pathways. In recent years, a growing body of evidence has shown the close association between DNA methylation and the onset and progression of inflammatory diseases. Alongside growing interest in preventive care and holistic well-being, TCM has gained increased recognition for its therapeutic potential. Because of its relatively mild side effects and practical effectiveness observed in clinics, TCM has gained favor among both patients and medical professionals. This increased attention has also sparked curiosity in the research community, prompting deeper investigations into how TCM might affect gene expression—particularly through mechanisms like DNA methylation. Although this area of study is still developing, early evidence points to the possibility that TCM may help regulate inflammation at the epigenetic level. However, some limitations remain in the current research. For example, the detailed molecular mechanism by which TCM regulates DNA methylation is not fully understood. Most studies mainly focus on the epigenetic level, and only a few have explored the specific molecular targets and signaling pathways involved; even these remain superficial. On the other hand, due to the complex composition of TCM formulations, it is hard to confirm active ingredients, and determining optimal dosages remain unresolved, bringing certain challenges to their clinical translation and standardized application.

Further research is necessary in regulating DNA methylation by using TCM in the treatment of inflammatory diseases. We are looking forward to better elucidating how TCM works at the molecular level. With ongoing advances in techniques like gene editing, proteomics, and transcriptomics, it is becoming more feasible to identify the key targets and signaling pathways involved. Moreover, these insights could contribute to treatments that are not only more effective but also better tailored to the specific mechanisms of disease. At the same time, carrying out large-scale, multi-center clinical studies will be crucial for moving TCM research closer to practical use in managing inflammatory diseases. Rigorous clinical trials will help validate the efficacy and safety of TCM in regulating DNA methylation, offering evidence for its integration into modern clinical practice. Additionally, modern pharmaceutical technology should be used to extract, separate and purify active components of TCM, enabling the development of standardized and quality-controlled formulations. Novel drug delivery systems, such as nanotechnology-based platforms, can also be used to enhance the bioavailability, targeting capability, and overall therapeutic efficacy of TCM interventions.

In summary, using TCM to regulate DNA methylation represents a promising research direction for the treatment of inflammatory disease. With ongoing, in-depth research and innovation, new opportunities may emerge for developing effective therapies, offering more treatment options and improved outcome for patients, and finally contributing to advancements to human health.

## Figures and Tables

**Table 1:** Chinese herbal compound regulates DNA methylation in the treatment of inflammatory diseases.

Chinese Herbal Compound	Composition of the Prescription	Model	Target	Regulation Mechanism	References
Xiaopi granules	Codonopsis Radix, *Codonopsis pilosula* (Franch.) Nannf. (root) Lilii Bulbus, *Lilium brownii* F. E. Brown var. *viridulum* Baker (Meaty scale leaves) Linderae Radix, *Lindera aggregata* (Sims) Kosterm. (tuberous root) Citri Fructus, *Citrus medica* L. (mature fruit) Salviae Miltiorrhizae, *Salvia miltiorrhiza* Bge. (roots and rhizomes) Notoginseng Radix Et Rhizoma, *Panax notoginseng* (Burk.) F. H. Chen (roots and rhizomes) curcumae rhizoma, *Curcuma zedoaria* (Christm.) Rosc. (rhizoma) Taraxaci Herba, *Taraxacum mongolicum* Hand. -Mazz. (whole plant) Herba Hedyotidis, *Oldenlandia diffusa* (Willd) Roxb. (whole plant)	SPF healthy male Wistar rats (chronic atrophic gastritis model)	Regulate DNA methyltransferase DNMT3B	Inhibition of DNMT3B expression; reverse abnormal DNA methylation; regulating cell proliferation and apoptosis; inhibition of inflammation	[64]
Liuwei Dihuang Pills	Rehmanniae Radix, *Rehmannia glutinosa* Libosch. (tuberous root) Corni Fructus, *Cornus officinalis* Sieb. et Zucc. (pulp) Dioscoreae Rhizoma, *Dioscorea opposita* Thunb. (rhizoma) oriental waterplantain rhizome, *Alisma orientale* (Sam.) Juzep. (tuber) Indian Bread, *Poria cocos* (Schw.) Wolf (sclerotium) Moutan Cortex, *Paeonia suffruticosa* Andr. (root bark)	Male ApoE^−^/^−^ mice (AS mice)	Regulation of *DNMT1* and *ER-α* gene methylation levels	Inhibition of DNMT1 expression; reverse *ER-α* gene methylation; up-regulate the expression of ER-α	[65]
Xuefu Zhuyu Capsule and Siji Sanhuang Capsule	Persicae Semen, *Prunus persica* (L.) Batsch (mature seed) Carthami Flos, *Carthamus tinctorius* L. (flower) Paeoniae Radix Rubra, *Paeonia veitchii* Lynch (root) Chuanxiong Rhizoma, *Ligusticum chuanxiong* Hort. (rhizoma) Scutellariae Radix, *Scutellaria baicalensis* Georgi (root) Phellodendri Amurensis Cortex, *Phellodendron amurense* Rupr. (bark) Fructus Gardeniae, *Gardenia jasminoides* Ellis (mature fruit) Rhei Radix Et Rhizoma, *Rheum palmatum* L. (roots and rhizomes)	Male ApoE^−^/^−^ mice (AS mice)	Regulation of DNA methylation and DNMT levels	Improve the level of DNA methylation; stable plaque structure; inhibition of inflammation	[66]
Shenyuandan formula	Astmgali Radix, *Astragalus membranaceus* (Fisch.) Bunge (root) Codonopsis Radix, *Codonopsis pilosula* (Franch.) Nannf. (root) Scrophulariae Radix, *Scrophularia ningpoensis* Hemsl. (root) Salviae Miltiorrhizae, *Salvia miltiorrhiza* Bge. (roots and rhizomes) Corydalis Rhizoma, *Corydalis yanhusuo* W. T. Wang (tuber) Eupolyphaga, *Eupolyphaga sinensis* Walker (The whole body of female insects) Hirudo, *Hirudo nipponica* Whitman (The whole body) Pheretima, *Pheretima aspergillum* (E. Perrier) (The whole body) Trichosanthis Fructus, *Trichosanthes kirilowii* Maxim. (mature fruit) Allii Macrostemonis Bulbus, *Allium macrostemon* Bge. (Bulb)	Clean-grade ApoE^−^/^−^ mice (AS mice)	Regulate DNMT1 level	Down-regulation of DNMT1; inhibition of inflammation	[67]
Danggui Shaoyao San	Angelicae Sinensis Radix, *Angelica sinensis* (Oliv.) Diels (root) Paeoniae Radix Rubra, *Paeonia veitchii* Lynch (root) Indian Bread, *Poria cocos* (Schw.) Wolf (sclerotium) Macrocephalae Rhizoma, *Atractylodes macrocephala* Koidz. (rhizoma) oriental waterplantain rhizome, *Alisma orientale* (Sam.) Juzep. (tuber) Chuanxiong Rhizoma, *Ligusticum chuanxiong* Hort. (rhizoma)	Eight-week-old ApoE^−^/^−^ mice (AS mice)	Regulation of methylation and DNMT1 levels	Inhibition of DNMT1 expression; reverse abnormal DNA methylation; inhibiting inflammation; reduce plaque area	[68]
ginseng and astragalus compound	Rehmanniae Radix, *Rehmannia glutinosa* Libosch. (tuberous root) Dioscoreae Rhizoma, *Dioscorea opposita* Thunb. (rhizoma) Corni Fructus, *Cornus officinalis* Sieb. et Zucc. (Mature pulp) Astmgali Radix, *Astragalus membranaceus* (Fisch.) Bunge (root) Ginseng Radix Et Rhizoma, *Panax ginseng* C. A. Mey. (root) Salviae Miltiorrhizae, *Salvia miltiorrhiza* Bge. (roots and rhizomes) Rhei Radix Et Rhizoma, *Rheum palmatum* L. (roots and rhizomes) Radix Trichosanthis, *Trichosanthes kirilowii* Maxim. (root)	Male spontaneous type 2 diabetic KKAy mice, aged 7–8 weeks (AS mice)	Regulation of gene DNA methylation	Inhibit inflammatory damage; protect vascular endothelial cells	[69]
Wutou decoction	Ephedrae Herba, *Ephedra equisetina* Bge. (herbaceous stem) Paeoniae Radix Rubra, *Paeonia veitchii* Lynch. (root) Paeoniae Radix Alba, *Paeonia lactiflora* Pall. (root) Astmgali Radix, *Astragalus membranaceus* (Fisch.) Bunge (root) Radix Rhizoma Glycyrrhizae, *Glycyrrhiza uralensis* Fisch. (roots and rhizomes) kusnezoff monkshood root, *Aconitum kusnezoffii* Reichb. (tuberous root)	Five-week-old female Wistar rats, weighing 130–150 g (CIA rats)	Regulates DNMT1	Inhibition of DNMT1 expression; reverse abnormal DNA methylation; reduce joint inflammation, inhibit synovial hyperplasia	[70]
Baihu Jia Guizhi decoction	Gypsum Fibrosum (ore) Anemarrhenae Rhizoma, *Anemarrhena asphodeloides* Bge. (rhizoma) Radix Rhizoma Glycyrrhizae, *Glycyrrhiza uralensis* Fisch. (roots and rhizomes) rice fruit, *Oryza sativa* L. (Dehulled seed kernels) Cmnamomi Mmulus, *Cinnamomum cassia* Presl (twig)	SPF SD rats, weighing 180–220 g (heat arthralgia model rats)	Regulate the expression levels of Methylation up-regulated gene *ACXT* and methylation down-regulated genes *AHCY* and *RPL3*	Regulating methylated gene expression; inhibit the production and release of inflammatory factors; improve foot swelling and pathological damage	[71]
Lang-Chuang-Ding Decoction	Rehmanniae Radix, *Rehmannia glutinosa* Libosch. (tuberous root) Trionycis Carapax, *Trionyx sinensis* Wiegmann (spinal brace) Artemisiae Annuae Herba, *Artemisia annua* L. (Whole grass on the ground) Herba Hedyotidis, *Oldenlandia diffusa* (Willd) Roxb. (whole plant) Centellae Herba, *Centella asiatica* (L.) Urban (whole plant) Paeoniae Radix Rubra, *Paeonia veitchii* Lynch (root) Coicis Semen, *Coix lacryma*-*jobi* L. var. *ma*-*yuen* (Roman.) Stapf (Mature seed kernel) Citri Sarcodactμlis Fructus, *Citrus medica* L. var. *sarcodactylis* (Noot.) Swingle (fruit) Rhizoma Cimicifugae, *Cimicifuga heracleifolia* Kom. (root) Radix Rhizoma Glycyrrhizae, *Glycyrrhiza uralensis* Fisch. (roots and rhizomes)	peripheral blood mononuclear cells. 1. Medicated Serum Preparation. 2. PBMCs Cultivation and Grouping. 3. Real-Time PCR. 4. MSP Assay for *CD70* Methylation.	Regulation of *CD70* gene promoter methylation level	Up-regulation of *CD70* gene promoter methylation; inhibition of *CD70* gene expression; inhibition of inflammation	[72]

**Table 2:** TCM monomer components regulate DNA methylation in the treatment of inflammatory diseases.

Chinese Herbal Medicinal Ingredient	Cell Type and Detection Index	Target	Regulation Mechanism	References
daphnetin	CIA rats’ synovial cells. 1. Cell viability assay. 2. Methylation specific PCR. 3. Flow cytometric analysis. 4. RNA extraction and gene expression analysis. 5. Apoptosis analysis by Annexin V/Propidium Iodide (PI) flow cytometry assay.	Regulate the expression of DNA methyltransferase DNMT1, DNMT3A and DNMT3B	Inhibition of DNA methyltransferase expression; inducing demethylation of pro-apoptotic genes; accelerating synovial cell apoptosis	[73]
gardenia jasminoides	RAW264.7 source foam cells. 1. Establishment of foam cell model. 2. Methylation immunoprecipitation combined with sequencing analysis.	Regulating abnormal DNA methylation genes	Bidirectional regulation of DNA methylation; inhibition of inflammation	[74]
periplogenin	HaCaT cells. 1. Immunofluorescence staining. 2. The level of ROS in cells was detected by flow cytometry. 3. The expression of the methylation-related gene P21 at the protein level was detected by Western blot.	To control the expression of DNA synthesis related enzymes and cell cycle regulatory proteins	Inhibition of DNA synthesis; reduce the expression of cell proliferation-related proteins; increase the level of p21 protein; promote apoptosis	[75]
hydroxycamptothecin	peripheral blood mononuclear cells. 1. Cell survival rate was assessed using the MTT assay. 2. RNA was extracted and analyzed by real-time quantitative PCR. 3. Genomic DNA was extracted and analyzed by methylation-specific PCR.	Regulation of DNMT1 and *p16* gene promoter region methylation	Inhibition of DNMT1 expression activity; induce *p16* gene demethylation; up-regulation of p16 protein expression	[76]
Sinomenine	A549 cell. 1. Cytotoxicity assay 2. Real-time PCR analysis 3. Bisulfite sequencing 4. Quantitative methylation-specific PCR (qMSP)	Regulate the DNA methylation level of *mPGES-1* promoter	Inhibit the expression level of *mPGES-1* gene and treat inflammation.	[77]
